# Advances in extracellular vesicle isolation methods: a path towards cell-type specific EV isolation

**DOI:** 10.20517/evcna.2023.14

**Published:** 2023-07-19

**Authors:** Adnan Shami-shah, Benjamin G Travis, David R Walt

**Affiliations:** ^1^Wyss Institute for Biologically Inspired Engineering, Harvard University, Boston, MA 02120, USA.; ^2^Department of Pathology, Brigham & Women’s Hospital and Harvard Medical School, Boston, MA 02120, USA.

**Keywords:** Extracellular vesicles, biomarkers, diagnostics, isolation methods, cell-type specific

## Abstract

Extracellular vesicles are small, heterogenous, phospholipid-rich vesicles that are secreted by all cells into the extracellular space. They play a key role in intercellular communication because they can transport a variety of biomolecules such as proteins, lipids, and nucleic acids between cells. As categorized by the International Society of Extracellular Vesicles (ISEV), the term EV encompasses different sub-types, including exosomes, microvesicles, and apoptotic bodies, which differ in their size, origin, and cargo. EVs can be isolated from biological fluids such as blood, urine, and cerebrospinal fluid, and their biomolecular content can be analyzed to monitor the progression of certain diseases. Therefore, EVs can be used as a new source of liquid biomarkers for advancing novel diagnostic and therapeutic tools. Isolating and analyzing EVs can be challenging due to their nanoscopic size and low abundance. Several techniques have been developed for the isolation and characterization of EVs, including ultracentrifugation, density gradient separation, size-exclusion chromatography, microfluidics, and magnetic bead-based/affinity methods. This review highlights advances in EV isolation techniques in the last decade and provides a perspective on their advantages, limitations, and potential application to cell-type specific EV isolation in the future.

## INTRODUCTION

Extracellular vesicles (EVs) are nanoscopic, membrane-bound, phospholipid-rich vesicles that are secreted by both prokaryotic and eukaryotic cells into the extracellular space^[1-3]^. EVs play a crucial role in intercellular communication because they can transport a wide variety of biomolecules such as proteins, lipids, nucleic acids, and metabolites between cells in a protected lipid shell^[2-4]^. Historically, EVs were regarded as cellular debris only responsible for clearing biomolecular byproducts^[5]^. Recently, there has been a tremendous rise in interest towards EVs because they have been established as a highly promising source of circulating biomarkers^[2,4]^.

EVs encompass several different types of vesicles, including exosomes, microvesicles, and apoptotic bodies, which differ in size and origin^[2]^. Exosomes are the smallest type of EVs (usually ranging from 30-100 nm) and are formed by endosomal cell sorting pathways in carefully orchestrated packing, budding, and fusion processes regulated by the ESCRT complex. In this pathway, intraluminal vesicles are formed by the invagination of multivesicular bodies. Various cargo molecules, such as proteins, nucleic acids, and metabolites, are packed inside these intraluminal vesicles, and they are eventually released as exosomes into the extracellular space through exocytosis^[6,7]^. Microvesicles are larger than exosomes (ranging from 100-1,000 nm), and are formed directly when a piece of the plasma membrane buds off into the extracellular space forming a vesicle^[6]^. The outward budding process involves the physical bending of the phospholipid membrane, which is tightly regulated by a series of calcium-dependent enzymatic steps involving scramblases, flippases, and aminophospholipid translocases^[6]^. Apoptotic bodies are another type of EVs (ranging from 1-5 µm), and are formed during apoptosis when the plasma membrane of dying cells becomes blebbed and forms vesicles^[2,6]^. The EV biogenesis process is discussed in greater detail in other reviews^[2,8,9]^.

EVs are involved in a wide range of pathophysiological processes, including immune responses^[10]^, inflammation^[10]^, cancer^[11]^, neuropsychiatric^[12]^, and neurodegenerative^[13]^ diseases. For example, microvesicles and exosomes released by immune cells can elicit an immune response by presenting antigens to other immune cells and initiating immune infiltration^[10]^. Moreover, exosomes have been shown to play a role in the spread of cancer cells, as they can transport oncogenic proteins and RNA to other cells, leading to tissue invasion and metastasis^[11,14]^. Additionally, there is increasing evidence suggesting that cancer-derived EVs carry differentially expressed molecular signatures than EVs from healthy tissues, suggesting the applicability of EVs for diagnostics^[1,4,11]^.

With the growing understanding that EVs comprise a new class of biomarkers, EVs are being increasingly investigated as diagnostic and therapeutic tools. EVs can be isolated from biological fluids such as blood, cerebrospinal fluid, urine, saliva, and breast milk, and their biomolecular contents may be analyzed to provide information about the disease progression of patients^[2]^. Additionally, EVs can be engineered to deliver therapeutic agents to specific cells or tissues, making them a potential delivery vehicle for drugs^[15]^. Other excellent reviews focus more thoroughly on the utility of EVs as sources of biomarkers in diagnostics^[16,17]^ and as delivery vehicles in therapeutic^[15,16,18]^ applications.

To be effectively utilized for biomedical applications, high-quality EV isolation techniques are required. The heterogeneity of EV size and their relatively low abundance compared to other contaminants in biological fluids make high-quality EV isolation a difficult challenge^[4,19]^. Many techniques have been developed for the isolation and characterization of EVs, including reduced solubility approaches, ultracentrifugation, density gradient separation, size-exclusion chromatography, microfluidics, and magnetic bead-based methods^[20]^. While these techniques have limitations, they have aided in advancing the field of EV research. This review discusses key EV isolation techniques focusing on their advantages, limitations, and potential areas of future development and provides a perspective on a path forward to achieving cell- or tissue-type EV isolation.

## CHALLENGES IN EXTRACELLULAR VESICLES ISOLATION

Depending on the biogenesis pathway, EVs can be of many different subtypes such as exosomes, microvesicles, and apoptotic blebs. Many of these EV subtypes tend to overlap in size, surface proteome, and cargo, making EVs broadly heterogeneous in nature^[1,2]^. Additionally, biofluids of interest for EV isolation contain varying compositions of proteins and non-EV lipid particles that contribute to the heterogeneity, complexity, and viscosity of the biofluid. For instance, analysis of blood serum and plasma estimates that there are roughly 10^11^ EVs and up to 10^16^ lipoproteins (LDLs/VLDLs/HDLs) per mL of blood plasma^[21,22]^. Furthermore, many biofluids, including blood plasma, serum, and cerebrospinal fluid (CSF), have a high abundance of albumin and matrix proteins. The lower relative abundance of EVs in these complex matrices can make high-purity EV isolation difficult, as both proteins and lipoproteins can co-isolate with the EVs^[21,23,24]^.

Apart from the problem of biological heterogeneity in EV isolation, many technical challenges persist that can also impact the overall yield and purity of EVs. Lipids have a high propensity to adhere to the walls of plastic tubes^[25]^. Additionally, researchers have shown that plastics can destabilize membranes by mechanical stretching^[26]^. Evtushenko *et al.* have shown that EV losses can reach 51% ± 3% when cell-culture-derived EVs are stored for 48 h at +4 °C in polypropylene Eppendorf tubes. Such significant EV loss could be reduced to 18%-21% by using Eppendorf Protein LoBind tubes or surface blocking with protein blockers^[27]^. Other critical challenges in EV isolation can be attributed to the methods employed for sample collection. Although EV counts seem to remain consistent over time in undisturbed samples, mild agitation designed to simulate blood handling during transportation results in a notable and artificial discharge of EVs derived from platelets^[28]^. Moreover, EVs tend to form EV-blood cell clusters over time, which can further impact EV isolation. Overall, the broad heterogeneity of EVs and their low relative abundance in biofluids, paired with technical inconsistencies associated with sample collection, pose a major challenge for EV isolation.

## CHALLENGES AND OPPORTUNITIES IN CELL-TYPE SPECIFIC EV ISOLATION

EV subpopulations that originate from specific types of tissue in the body carry the biomolecular signature of their cells of origin. EVs are rich sources for a new form of 'liquid biopsy' because they have a lipid shell comprising transmembrane proteins on their surface and cargo proteins, nucleotides, and metabolites inside of them^[2-4]^. They may provide detailed molecular profiling of cells from hard-to-access organs (e,g., brain), which would otherwise require an invasive biopsy. This can open diagnostic opportunities for diseases that cannot be detected presently with blood tests. Additionally, cell-type specific EVs could pave a pathway to study disease etiology by monitoring progressive changes at the cellular and molecular levels in a minimal to non-invasive way.

One of the major barriers to cell-type specific EV isolation is the rare and very low abundance of cell- and tissue-type specific EVs in human biofluids. For instance, blood plasma is a repository of EVs from many different cells and tissues. *Auber et al.* used EV RNA cargo profiling to estimate that, of the total EVs in healthy blood plasma, around 99% originate from platelets, erythrocytes, and white blood cells. Less than 1% originate from solid tissues^[29,30]^. Furthermore, when the researchers examined the EV-to-parental cell type ratio on a cell-specific level, they observed a they observed a significant range spanning four orders of magnitude. That range extended from 0.13 ± 0.1 erythrocyte-derived EVs per erythrocyte to 1.9 ± 1.3 × 10^3^ monocyte-derived EVs per monocyte^[29]^. Therefore, this broad range in the number of EVs released per cell between different cell types, along with existing contaminants in the biofluids, make isolating cell-type specific EVs a monumental challenge.

While conventional techniques have allowed for EV isolation from various biofluids, additional advances are needed to achieve the separation of rare EV subtypes. Understanding the benefits and limitations of these techniques further paves a pathway towards utilizing them for sub-type-specific EV isolation. This review provides a brief overview of various techniques, describes their challenges and limitations, and adds a potential pathway towards cell-type specific EV isolation.

## EV ISOLATION TECHNIQUES

### Ultracentrifugation

Ultracentrifugation has been one of the most widely used methods for EV isolation from complex biological samples^[31]^, which is based on the principle of sedimentation, where EVs are separated from other biomolecules based on their shape, size, and density^[20]^. Ultracentrifugation involves first centrifuging a sample at a low speed of up to 2,000 xg to remove large debris and dead cells. The resulting supernatant is then centrifuged at 16,500 xg or less to pellet large apoptotic bodies^[31]^. After removing these larger species, the supernatant is placed in an ultracentrifuge and spun at high speeds, typically around 100,000 xg or greater, for several h^[32]^. The high g-force generated from the centrifugation separates different components of the biofluid with the EVs pelleting at the bottom of the tube. The speed and duration of centrifugation depend on the size and density of the EVs being isolated from the samples. The pellet can be washed and resuspended in buffer for downstream analyses or long-term storage^[20,32,33]^.

Ultracentrifugation has been a broadly used technique for EV isolation due to several advantages-particularly its adaptability to large volumes of samples. This attribute helps increase EV yield and makes the approach well-suited for studies where large sample volumes are available, such as cell culture media or easily accessible biofluids^[20,34]^. For example, urine has been used to successfully differentiate patients with prostate cancer from healthy controls using EVs obtained by ultracentrifugation^[35]^. Furthermore, ultracentrifugation is also relatively inexpensive compared to other methods, such as immunoaffinity isolation.

While ultracentrifugation is a relatively simple technique that does not require any complex instrumentation, there are several limitations to this method. Ultracentrifugation may result in partial EV aggregation and degradation as the high centrifugal force required may lead to artificial fusion of smaller EVs and fission of larger EVs. This not only limits the ability of ultracentrifugation to isolate EVs of uniform size, but can also lead to the loss of some of their original biomolecular contents^[33]^. Additionally, co-sedimentation of non-EV biomolecules, such as lipoproteins and protein aggregates with similar buoyancy to that of EVs, can cause yield and purity problems^[20]^. These disadvantages are only amplified when attempting to isolate EVs from sample matrices with higher viscosities, making ultracentrifugation a less viable option in some biofluids such as plasma^[20,36,37]^. Moreover, lengthier spin times also make this technique less efficient and translatable to a clinical setting. Therefore, while ultracentrifugation may still be a viable option in the clinic when working with large volumes of dilute samples, this approach is likely incompatible with the analysis of low-abundance EV subtypes that are present in complex biofluids.

### Density gradient centrifugation

Density gradient centrifugation (DGC) is a method that is commonly used in conjunction with ultracentrifugation to isolate extracellular vesicles (EVs) from biological fluids^[23,38]^. DGC involves the use of a density gradient, which is created by layering solutions with a range of different densities in a tube, such as sucrose or iodixanol (OptiPrep)^[39]^. The process starts by filtering or centrifuging the samples to remove debris and large particles that could interfere with the EV isolation. This process also dilutes the samples allowing them to pass through the thick gradient during the prolonged centrifugation times required. The sample is then layered on top of the density gradient and centrifuged at high speed for many hours, depending on the type of biofluid, to separate the different components of the samples^[23,38]^. Exosomes have a characteristic buoyant density of 1.10-1.19 g/mL on sucrose density gradients^[40]^. Post centrifugation, samples are collected from each density fraction of the gradient and EVs are found in the denser fractions. Typically, a refractometer is used to measure the density using the refractive index of each fraction^[23]^.

DGC has been a useful technique for many cell-biological applications, including isolating organelles^[41]^ and determining the oligomeric states of a protein^[42]^. While it has been a widely applied technique in the field of EV research, it has some limitations for EV isolation^[20]^. Depending on the medium being used, DGC requires specialized equipment and can be expensive, low throughput, and time intensive (more than 18-hour spin to reach density equilibrium)^[23,37,43,44]^. Because of the long time required, DGC is not highly applicable to use in a clinical setting or efficient enough for biomarker discovery^[24]^. While DGC yields EVs with a relatively high purity compared to ultracentrifugation, higher-order protein aggregates and lipoproteins with similar densities to EVs persist as contaminants^[20,45]^. For example, while KBr-density gradient ultracentrifugation on plasma successfully removes VLDLs from EV fractions, HDLs persist at a ratio of EVs to HDLs estimated to be as high as 1:100 by TEM^[45]^. Impurities can also arise due to the improper setting up of the gradient, causing intermixing of the gradient interfaces and fraction mixing. All these issues can contribute to an overall low yield and EV contamination, which should be considered when optimizing DGC isolation^[20]^. Even with these limitations, DGC has been an important technique in EV research, providing utility both as an orthogonal validation tool for evaluating subtype EV markers and as a method for disease-state bulk EV analysis in biofluids such as urine^[23,46]^.

### Size Exclusion Chromatography

**Figure 1:**
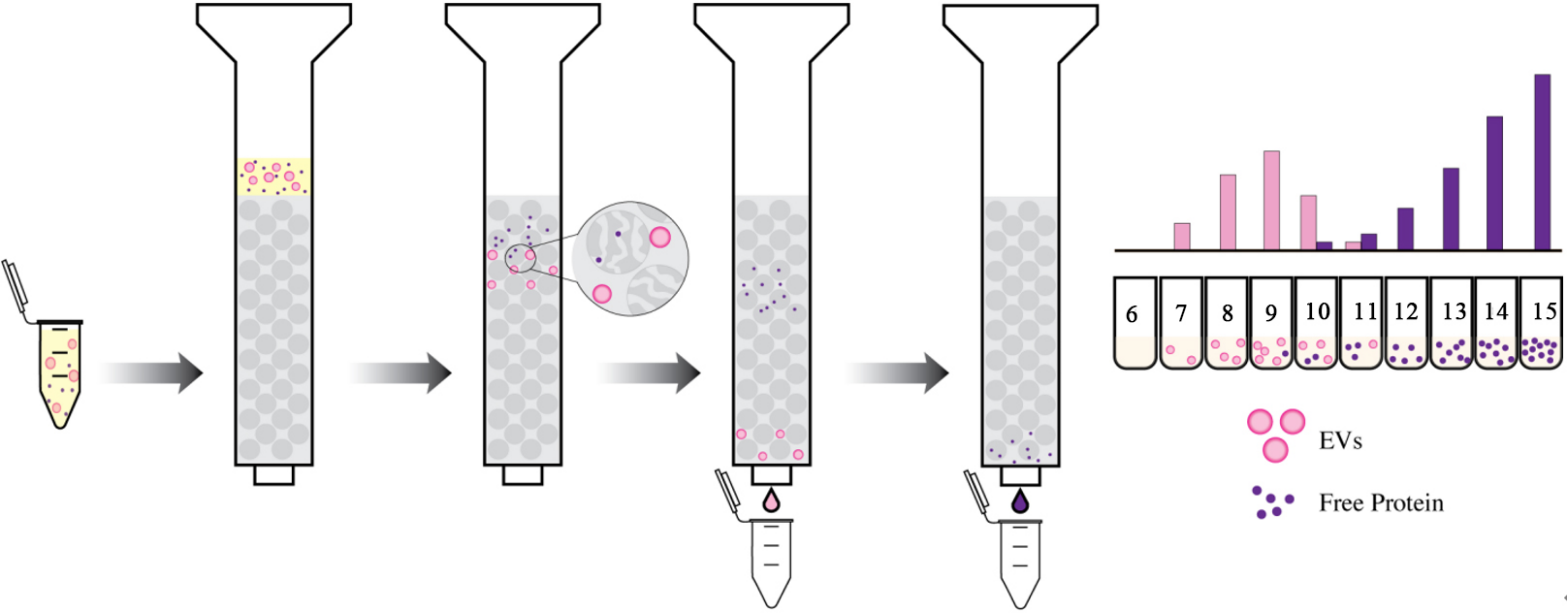
Size Exclusion Chromatography (SEC) separates molecules based on size. Simplified diagram of SEC showing separation of EVs (pink) from soluble free proteins (purple).

Size Exclusion Chromatography (SEC), conventionally referred to as gel filtration, is a method for isolating biological species such as proteins or vesicles, based on their hydrodynamic radius, which is equivalent to the apparent size of the solvated species^[20,47]^. EVs can be separated from soluble proteins based on their ability to be excluded or pass through pores of different sizes in a chromatography column [Figure 1]. To isolate EVs using SEC, sample is passed through the stationary phase of a chromatography column that is packed with beads of a porous material, such as agarose (Sepharose)^[24,48]^, allyl dextran (Sephacryl)^[48]^, or cross-linked dextran (Sephadex)^[48]^. The beads have pores of different sizes, and as the sample passes through the column with the flow of the mobile phase, the EVs are separated based on their size. Smaller EVs get trapped in the pores of the resin for longer times, so they are eluted from the column later, while larger EVs avoid the pores of the resin and mostly pass through the larger channels, so they are eluted earlier. The separated EVs are collected in fractions and can then be analyzed or used for further experimentation^[20,24,47,48]^.

The separation of EVs from various biological fluids, including CSF and plasma, can be optimized based on the column length, size, type of resin, and flow rate of the mobile phase^[24]^. These parameters can further influence the yield and purity of the isolated EVs; therefore, the protocol must be thoroughly optimized based on the application. SEC has been adapted as a single-step isolation system and modified over the years for greater purity and yield^[47]^. This technique aids in separating small from large EVs and from non-EV soluble protein contaminants in samples, with an average 20 min processing time, resulting in time and cost-effective, pure, intact, and functional EV retrieval l^[47,49]^. SEC can also be scaled up and automated, making it a high-throughput method for EV isolation. It can be performed with varying sample volumes, making it useful for isolating EVs from samples with limited volume availability (e.g., CSF). Additionally, due to the use of gravity as an isolation principle for SEC rather than high g-forces for ultracentrifugation or DGC, the isolated EVs are of superior integrity with intact vesicular properties and higher quality^[47,49,50]^.

A key drawback to this technique is that SEC cannot effectively differentiate between microvesicles and exosomes of similar size^[47]^. Furthermore, limitations include the need for specialized resins, columns, and co-elution of similarly sized lipoproteins and large protein aggregates^[37]^. Additionally, the dilution of sample created by the mobile phase of SEC makes isolating subpopulations of EVs from low-concentration and small-volume samples a challenging task. Despite this limitation, SEC has been shown to outperform the ultracentrifugation and precipitation method described below in terms of purity and yield in both plasma and CSF, making it a powerful isolation technique for EV research^[24]^.

### Dual Mode Chromatography

Biological fluids, such as plasma, are often heavily contaminated with plasma lipoprotein particles that can outnumber EVs more than 10^4^-fold^[51]^. Due to the overlap in size with EVs, lipoproteins cannot be completely removed from biofluids using standard SEC. Dual mode chromatography is a powerful new emerging technique for isolating higher purity extracellular vesicles (EVs) from biofluids by depleting charged lipoproteins^[51,52]^. The method involves the use of two different chromatography methods in sequence, each designed to remove specific types of contaminants and purify EVs based on their physical, chemical, and electrostatic properties.

**Figure 2:**
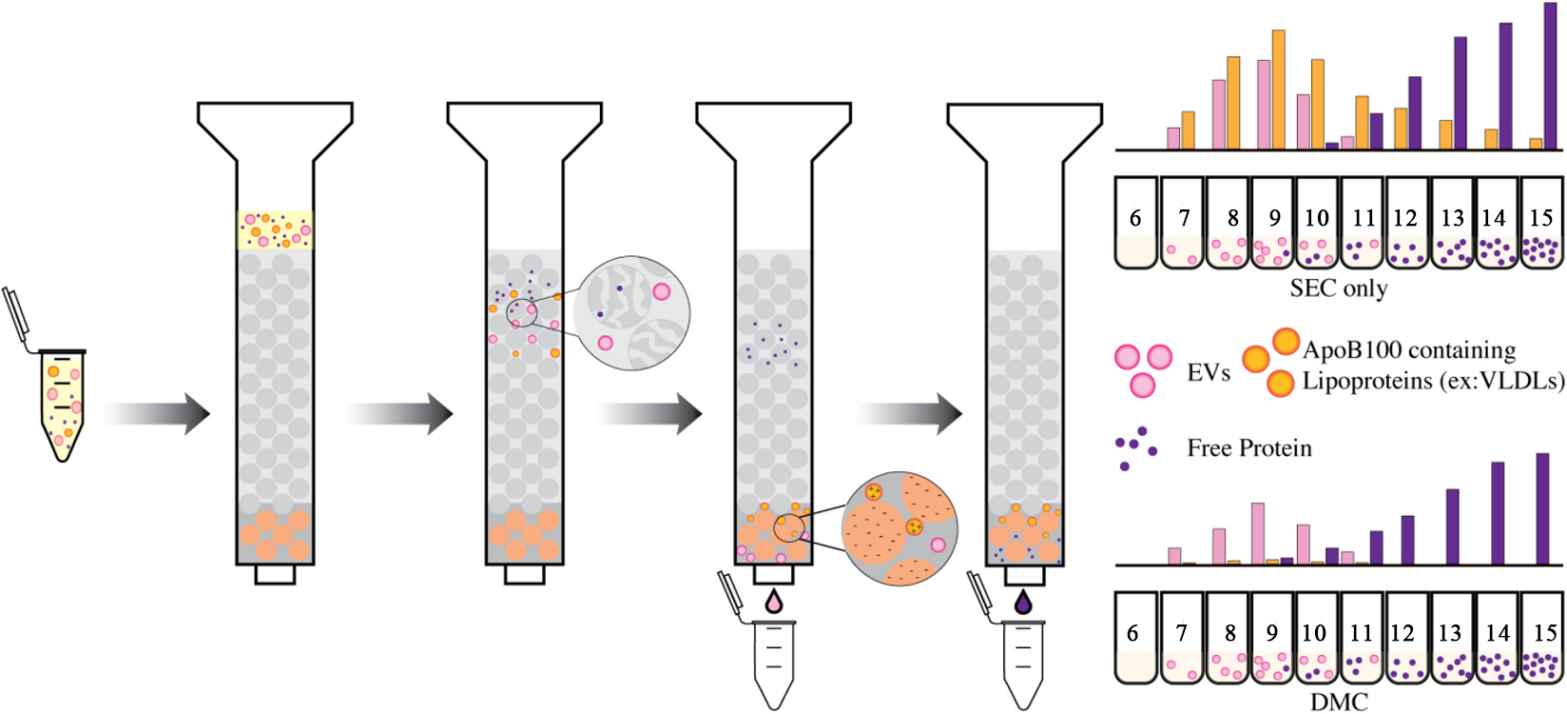
Dual Mode Chromatography (DMC) separates molecules based on both size and charge interactions. The diagram shows the comparison of methods for isolation of EVs (pink) from free proteins (purple) and charged lipoproteins (orange). Note that EV isolation using only SEC gives higher EV yields, but they are often contaminated with lipoprotein co-isolates in a similar size range, while using DMC, the EVs yield is lower but free of lipoprotein contaminants.

The first step in the process typically involves size exclusion chromatography (SEC), as described above, which traps smaller analytes such as soluble proteins, aggregates, and HDL in the resin based on differential retention times^[51]^. The second step in the process is ion exchange chromatography (IEC), which separates particles based on charge. Given that the surface of EVs is mostly negatively charged, a cation exchange resin is typically used. This resin carries a net negative charge that retains the positively charged ApoB100-containing VLDL particles, while allowing EVs to be eluted^[51]^. When assembling the column, the SEC resin (described above) is placed directly on top of the IEC resin [Figure 2]. The IEC layer is often comprised of Fractogel EMD SO_3_^-^ resin capable of removing up to 70-fold more ApoB100 particles than other similarly charged resins^[51]^.

A study comparing the ability of SEC and DMC to isolate EVs from lipoprotein particles in plasma found that while the techniques remove HDLs from EV containing fractions with equally high efficacy (~ 97% removal efficiency of ApoA1 for both SEC and DMC), the EV fractions of DMC filtered plasma retained only 0.4% of the original ApoB100 plasma content compared to SEC’s 25% retention rate^[51]^. While this approach allows for the isolation of higher purity EVs from biological fluids by removing non-EV particles, EV recovery ratios of a pure EV standard using SEC and DMC are 0.78 and 0.34, respectively^[51]^. This decrease in yield for DMC is likely due to the longer column retention times required^[51,52]^. Furthermore, the tradeoff between yield and purity with DMC must be considered along with EV abundance when determining if this isolation technique is appropriate for analyzing different EV subpopulations. Overall, DMC is a powerful new emerging technology that provides one more tool for navigating the highly heterogenous composition of biological samples.

### Magnetic Bead-based/Immunoaffinity isolation methods

Magnetic bead-based methods isolate extracellular vesicles (EVs) using specific antibodies or affinity ligands coated on the surface of magnetic beads^[20,47]^. There are several magnetic bead-based approaches for EV isolation, including magnetic bead-based immunoaffinity chromatography and magnetic bead-based ligand affinity chromatography^[20,53]^.

**Figure 3:**
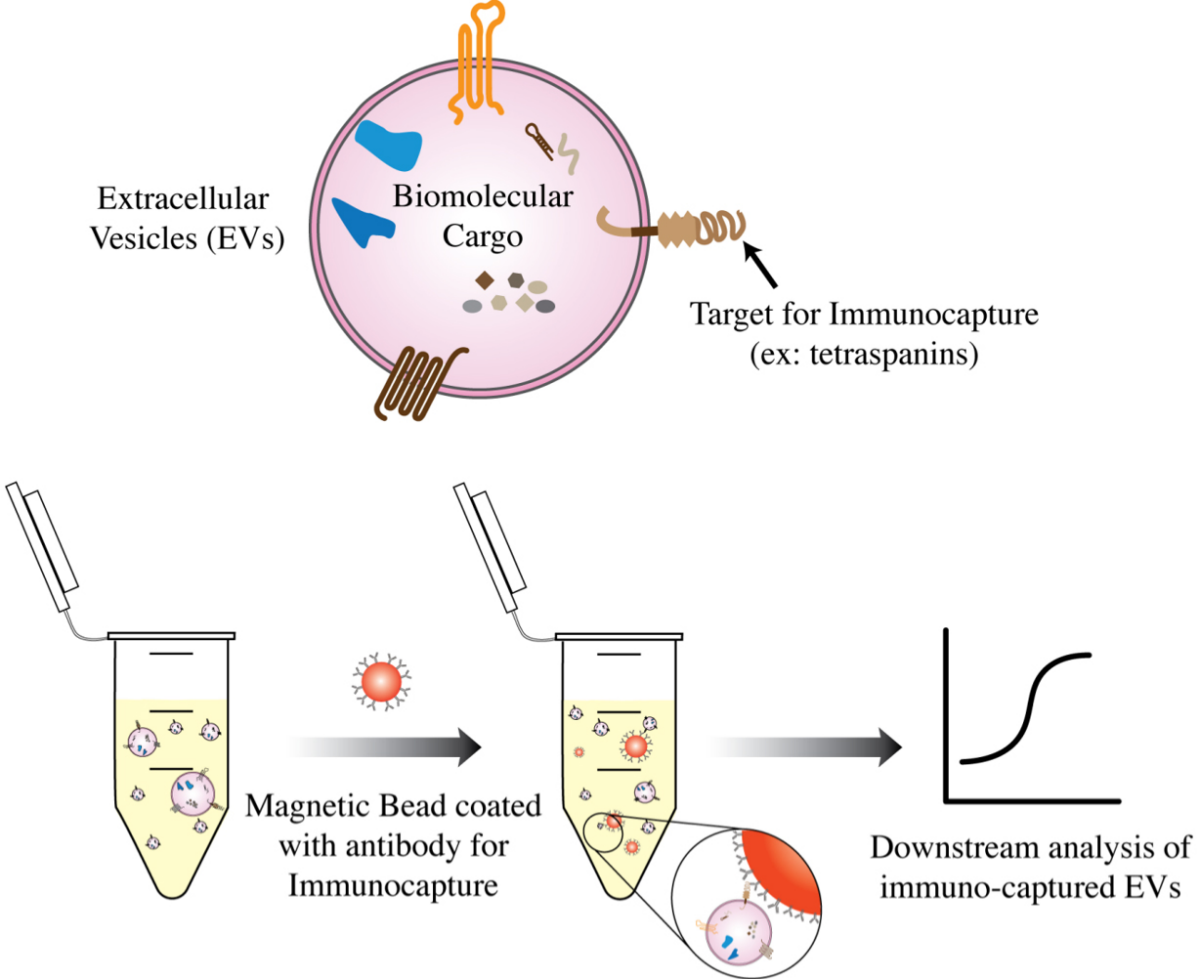
Magnetic beads coated with antibodies against surface markers on the EVs can be used to immunocapture EVs of a certain type for downstream analysis.

In magnetic bead-based immunoaffinity chromatography, magnetic beads are coated with antibodies that bind to specific proteins or other biomolecules on the surface of the EVs^[20,47,53]^. The sample is then incubated with the magnetic beads, and the EVs are captured by the beads through specific binding interactions [Figure 3]. For instance, antibodies against generic EV surface markers, specifically the tetraspanins CD9, CD63, and CD81, have been used conventionally to perform bulk immunoaffinity EV isolation^[47]^. In magnetic bead-based ligand affinity chromatography, the magnetic beads are coated with ligands that specifically bind to receptors or lipids such as phosphatidylserine on the surface of the EVs^[54,55]^. The sample is incubated with the magnetic beads, and the EVs are affinity captured by the beads through ligand-receptor interactions. In both cases, the magnetic beads containing the captured EVs are then separated from the rest of the biomolecules in the sample using a magnetic field. The beads are then washed with buffers to reduce nonspecific interactions and the EVs on the beads can be subjected to further downstream analysis.

There are several advantages to using magnetic bead-based methods for isolating EVs. Unlike the other methods listed in this review, magnetic bead-based immunoaffinity isolation allows for the capture of EVs based on specific biomolecules or receptors, which is useful for isolating subpopulations of cell-type specific EVs^[20,47,53]^. Additionally, magnetic bead-based methods may be automated to achieve high throughput EV isolation, and can also be useful for isolating EVs from a low sample volume because the approach is highly targeted^[53]^. However, these methods can have some limitations including the requirement for specialized equipment, the high cost of antibodies, and the requirement for high-affinity antibodies or ligands to immunocapture certain populations of EVs. Additionally, because this is a targeted approach, a lack of good antibodies could be a bottleneck for the successful use of this method.

Nonspecific binding is another major drawback of magnetic bead capture. Both the adhesion of sample contents to the magnetic bead’s surface and off-target antibody binding can result in impurities and can lead to false conclusions about the contents of EVs in downstream analyses. Lipids tend to adhere to surfaces nonspecifically^[27]^. Additionally, there are many challenges associated with verifying the low of abundance internal cargo biomolecules detected after immunocapture of EVs. Hence, nonspecific binding is especially problematic when attempting to isolate low-abundance EV subpopulations. Therefore, careful reagent validation and optimization of bead surface chemistry is a crucial step for bead-based immunocapture of EVs. If successfully validated, this technique provides a powerful platform for rapid immunoenrichment of both bulk EVs and EV subpopulations and is highly adaptable to the clinic.

### Microfluidics-based methods

Microfluidics enable the manipulation and analysis of small volumes of fluids at the microscale. There are several microfluidic methods and devices for EV isolation, including microfluidic filtration, affinity isolation^[56,57]^, and dielectrophoresis (DEP)^[58,59]^.

In microfluidic filtration, EVs are separated from a sample using a microfluidic device with an array of nanoporous *in-situ* filters pre-designed for a specific size. A low volume of sample is passed through the filters allowing small EVs to pass through, while other components of the sample, such as cells and debris, get trapped^[56,57]^. The requirement for frequent filter and channel de-clogging, even with small sample volumes, is a major drawback of this technique. In microfluidic affinity isolation, the sample flows through polydimethylsiloxane (PDMS) microfluidic chips that are functionalized with affinity reagents, such as antibodies. ExoChip™ is such a microfluidic affinity system that exploits both the affinity and filtration capabilities of microfluidics. It consists of an array of microfluidic channels coated with a specific binding agent, such as an antibody against the EV surface marker CD63^[60]^. When a sample containing EVs is passed through the chip, the EVs are captured by the binding agent and retained, while other molecules pass through. ExoChip™ has been shown to be highly efficient at isolating EVs, with a reported recovery rate of over 90% for tetraspanins^[60]^. It is also fast, with a processing time of less than 30 min. In addition, it is relatively low-cost and easy to use, making it a promising tool for EV isolation and analysis. In microfluidic DEP, EVs are separated from a sample using an alternating current electrokinetic (ACE) microarray chip^[58,59]^. The sample is subjected to an alternating current, and the EVs are concentrated into high-field regions within a short time scale while other components of the sample are separated based on their dielectric properties.

There are several advantages to using microfluidics-based methods for EV isolation. They have the potential to be automated and miniaturized, making them a high-throughput and portable method for EV isolation^[20,47]^. Additionally, sample volumes as low as 10 µL can be used for isolation. Some limitations of this technique include the requirement for specialized equipment, low sampling efficiency, frequent channel clogging, and the need for antibodies with high affinity and specificity. Due to such limitations, microfluidics-based methods are not yet considered standard methods for EV isolation^[20,47]^.

### Reduced solubility approaches

EV precipitation by attenuating solubility is one of the earliest conventional approaches used to isolate EVs from a variety of complex biofluids. One such method includes using hydrophilic polymers, such as dextran or polyethylene glycol (PEG)^[61]^. These polymers, when added to a sample biofluid containing EVs, will form a complex with EVs due to their negative charge. The complex can then be precipitated by adding a neutralizing agent, such as cationic beads or an excess of cationic protein. The EVs can then be recovered from the precipitate by centrifugation at a low speed (< 1500 xg) or by filtration^[20,47]^. There are several commercial PEG-based EV precipitation kits available, including ExoQuick®, ExoQuick® ULTRA (System Biosciences), ExoPrep™ (HansaBioMed), Total Exosome Isolation Kit (Invitrogen), and miRCURY™ (Qiagen) that can be used based on the application, type, and volume of biofluid being used as a starting material^[20]^. Any approach should be thoroughly optimized, as the properties of the hydrophilic polymer and the neutralizing agent can affect the efficiency of EV isolation.

While bulk sample yields may be higher relative to other isolation techniques, purities are often compromised when utilizing these reduced solubility approaches. For instance, in a study conducted by Garcia-Romero *et al.*, a PEG-based EV precipitation method led to the highest yield for both precipitated EVs along with protein contents from the sample^[62]^. While another study by Gamez-Valero reported similar results with high protein and EV yields using PEG-based precipitation methods, downstream cryo-EM analysis of the precipitants did not detect any EVs, and molecular profiling of the precipitants showed EVs that were only CD9 + /CD63-/CD81-suggesting the presence of other bulk contaminants confounding EV isolates^[63]^. Thus, PEG-based polymers are nonspecific reagents for EV precipitation that is often accompanied by other non-EV precipitants such as albumin, lipoproteins, and immunoglobulins^[47,64]^. These limitations make reduced solubility approaches ineffective for cell-type specific EV isolation. Importantly, efficient processing time, simplicity, and low cost make this method particularly attractive for crude and rapid EV isolation. Researchers may want to use this method for preliminary analysis before pursuing other isolation methods that could help improve yield and purity.

## A PATH TOWARDS CELL-TYPE SPECIFIC EV ISOLATION

With continued advances in EV isolation techniques on many fronts, cell-type specific EV isolation is more possible than ever. However, several challenges persist^[65]^ [Table 1]. Given that every cell type in the human body secretes EVs into biofluids, relative proportions of cell-type specific EVs can depend partly on the abundance of that cell type. Additionally, EV secretion rate has been shown to differ drastically between cell types in cultured cells^[29]^. While cell-type specific EV secretion rates have yet to be determined in vivo, both low cell-type abundance and low basal EV secretion rates make some cell-type specific EVs difficult to isolate using conventional technologies due to their low relative concentrations in biofluids. Furthermore, in plasma, EVs originating from tissues have been estimated to account for less than 1% of all EVs, as opposed to the 99.8% generated from hematopoietic cells^[30]^. Because EVs act as antigen-presenting vesicles with various membrane-bound proteins, it may be possible to capitalize on this inherent property for targeted EV isolation^[2]^. Therefore, targeted immuno-enrichment or affinity isolation-based methods are an essential tool in using tissue-derived cell-type specific EVs for 'liquid biopsy', as the analyses of their biomolecular contents can only be successful if their relative abundance is selectively increased.

**Table 1:** A summary table describing the advantages and limitations of various techniques for isolation of EVs and their applicability for cell-type specific isolation of EVs

**Separation Approach**	**Ultra-centrifugation**	**DGC**	**SEC**	**DMC**	**Bead-Based Immuno-affinity**	**Microfluidics**	**Reduced Solubility**
Maximum sample volume	H	M	M	M	L	L	L/M
Time	M	H	L	L	M	L	L/M
Cost	L	M	L	L/M	H	L/M	M
Yield	L/M	M	H	M	M	M	H
Purity	L	M	M	H	M*	M*	L
Throughput capacity	L	M	M/H	M/H	M/H	H	M/H
Applicability to cell-type specific EV isolation	L	L/M	H	H	H	H	L

L: Low; M: Medium; H: High; EVs: Extracellular vesicles; DGC: Density gradient centrifugation; SEC: Size Exclusion Chromatography; DMC: Dual Mode Chromatography. *Can be antibody-dependent.

To advance affinity-based techniques, a crucial step is to identify cell-type specific transmembrane proteins that can be used as handles for immuno-isolation. Proteins in the human body can exist in multiple isoforms due to alternative splicing. Consequently, in many instances, a particular transmembrane protein can have both transmembrane as well as secreted isoforms. Hence, an antibody used to capture the extracellular domain for EV immuno-isolation could capture both the transmembrane and secreted forms and confound results^[23]^. Therefore, selection of cell-type specific markers based solely on transcript or gene enrichment analysis in a particular cell type is not sufficient. Thorough isoform analysis should be performed to aid in the rational selection of both immuno-isolation targets and antibodies that ideally bind epitopes present only in the transmembrane isoform but not in the secreted isoforms. With the growing availability of human proteome, secretome, and single-cell transcriptomics databases, our rational approach to select targets should improve over time^[3]^.

Immunoaffinity techniques can be paired with other technologies discussed in this review to create hybrid EV isolation procedures that increase the efficacy of immuno-enrichment [Table 1]. For example, preliminary sample processing with SEC or DMC can help pre-purify EVs by removing secreted target isoforms that may interfere with EV immunocapture, and/or reduce the concentration of lipoproteins that may adhere to surfaces nonspecifically. Additionally, immunoaffinity paired with microfluidics could offer several advantages. Using microfluidic hybrid techniques, EVs could be selectively filtered based on their size, shape, and surface biomarkers, which may allow for the isolation of even rarer subtypes. Regardless of the technique chosen, these complementary purification steps must be carefully optimized to maximize EV yield, given the low abundance of some cell-type specific EVs. Moreover, even with effective preliminary sample processing, the results of cell-type specific EV immunocapture can be extremely misleading if rigorous validation of the immuno-enrichment protocol is not performed to account for nonspecific binding. This is a crucial step in any cell-type specific EV isolation process that relies on immunoaffinity or utilizes surfaces to which lipid particles may adhere. However, even with careful optimization, techniques such as ultracentrifugation that can compromise the integrity of the EV membrane are not well suited for cell-type specific EV isolation, as EV fusion and aggregation could interfere with the analysis of specific internal cargo. Overall, rigorous target analysis paired with emerging isolation techniques should enable cell-type specific isolation and pave the path towards a new era of EV research and clinical applications.

## OUTLOOK

The growing understanding of extracellular vesicle biology, paired with advances in EV isolation tools and techniques [Table 1], has significantly propelled the field of EV research in the last decade^[66]^. EVs are a new class of biomarkers that are being exploited for their potential in both diagnostics and therapeutics, especially for diseases that cannot undergo biopsy easily. Due to their heterogeneity in size and the diversity of the EV surface proteome, the optimal EV isolation strategy is mostly based on the type, variety, and volume of the biofluid being used as the starting material^[21]^. A lack of harmonization in EV isolation methods has resulted in inconsistencies in EV research. With the development of many novel EV isolation techniques in the last decade, standardized isolation methods based on the desired yield and purity of EVs would help tackle some of the inconsistencies in the field of EV research.

Furthermore, while gene expression has been the initial strategy to select a cell-type specific EV isolation target, rigorous bioinformatic analyses must be performed to analyze the target structure, isoforms, and topology to minimize nonspecific interactions and misleading results due to reagent cross-reactivity. Systematic generation of data from such studies can be of great value for designing better studies in the future. Those studies would further help create novel diagnostics and therapeutic opportunities. In addition, rigorous binding reagent validation is required to ensure the EV subtype is properly targeted. Most importantly, the advancement of EV isolation tools and technologies, standardization of data analysis pipelines, accessibility of isolation protocols, and high technical rigor are all required to harness the full potential of the next generation of extracellular vesicle biology research.

## DECLARATIONS

### Copyright

©The Author(s) 2023.
